# Google DeepMind and healthcare in an age of algorithms

**DOI:** 10.1007/s12553-017-0179-1

**Published:** 2017-03-16

**Authors:** Julia Powles, Hal Hodson

**Affiliations:** 1 Faculty of Law and Computer Laboratory, University of Cambridge, Cambridge, UK; 2 The Economist Newspaper, London, UK

**Keywords:** Artificial intelligence, Clinical care, Consent, Data protection, Machine learning, Power, Privacy, Regulation

## Abstract

Data-driven tools and techniques, particularly machine learning methods that underpin artificial intelligence, offer promise in improving healthcare systems and services. One of the companies aspiring to pioneer these advances is DeepMind Technologies Limited, a wholly-owned subsidiary of the Google conglomerate, Alphabet Inc. In 2016, DeepMind announced its first major health project: a collaboration with the Royal Free London NHS Foundation Trust, to assist in the management of acute kidney injury. Initially received with great enthusiasm, the collaboration has suffered from a lack of clarity and openness, with issues of privacy and power emerging as potent challenges as the project has unfolded. Taking the DeepMind-Royal Free case study as its pivot, this article draws a number of lessons on the transfer of population-derived datasets to large private prospectors, identifying critical questions for policy-makers, industry and individuals as healthcare moves into an algorithmic age.

## Introduction

A key trend in contemporary healthcare is the emergence of an ambitious new cadre of corporate entrants: digital technology companies. Google, Microsoft, IBM, Apple and others are all preparing, in their own ways, bids on the future of health and on various aspects of the global healthcare industry.

This article focuses on the Google conglomerate, Alphabet Inc. (referred to as Google for convenience). We examine the first healthcare deals of its British-based artificial intelligence subsidiary, DeepMind Technologies Limited,^1^ in the period between July 2015 and October 2016.^2^ In particular, the article assesses the first year of a deal between Google DeepMind and the Royal Free London NHS Foundation Trust, which involved the transfer of identifiable patient records across the entire Trust, without explicit consent, for the purpose of developing a clinical alert app for kidney injury. We identify inadequacies in the architecture of this deal, in its public communication, and in the processes of public sector oversight. We conclude that, from the perspective of patient autonomy, public value, and long-term competitive innovation, existing institutional and regulatory responses are insufficiently robust and agile to properly respond to the challenges presented by data politics and the rise of algorithmic tools in healthcare.

The article proceeds in three main sections. The next two sections document comprehensively how the DeepMind deals proceeded, drawing attention to the disclosures and omissions in how data handling was communicated, justified and, ultimately, scrutinized in public. Section 2 discusses the chronology, formal contractual basis, and stated clinical motivation underlying the Royal Free deal, highlighting the delayed revelation of the nature and scale of patient data involved. Section 3 explores DeepMind’s broader ambitions in working with the NHS and the lack of *ex ante* discussions and authorizations with relevant regulators. It also elaborates on the problematic basis on which data was shared by Royal Free, namely, the assertion that DeepMind maintains a direct care relationship with every patient in the Trust. Section 4 then lays out the lessons that can be drawn from the case study as a whole, assesses at a high level the data protection and medical information governance issues, and then turns to transparency, data value, and market power.

## A startup and a revelation

In July 2015, clinicians from British public hospitals within the Royal Free London NHS Foundation Trust approached Google DeepMind Technologies Limited, an artificial intelligence company with no experience in providing healthcare services, about developing software using patient data from the Trust [1]. Four months later, on 18 November 2015, [2] sensitive medical data on millions [3] of Royal Free’s patients started flowing into third-party servers contracted by Google to process data on behalf of DeepMind [4].

Royal Free is one of the largest healthcare providers in Britain’s publicly funded National Health Service (NHS). The NHS offers healthcare that is free at the point of service, paid for through taxes and national insurance contributions. Beloved in the UK, the NHS is a key part of the national identity.

DeepMind publicly announced its work with Royal Free on 24 February 2016 [5]. No mention was made of the volume or kind of data included in the transfer—millions of identifiable personal medical records. DeepMind said it was building a smartphone app, called ‘Streams’, to help clinicians manage acute kidney injury (AKI). AKI has outcomes ranging from minor kidney dysfunction through to dialysis, transplant, and even death, and is linked to 40,000 deaths a year in the UK [6, 7]. The app, DeepMind claimed, would not apply any of the machine learning or artificial intelligence techniques (effectively, statistical models built using powerful computing resources over large corpora of granular, personalized data [8]) for which it is renowned, and would act as a mere interface to patient medical data controlled by Royal Free [9]. Why DeepMind, an artificial intelligence company wholly owned by data mining and advertising giant Google, was a good choice to build an app that functions primarily as a data-integrating user interface, has never been adequately explained by either DeepMind or Royal Free.

### Contractual foundations vs public relations

Throughout the whole first phase of the deal, through to October 2016, DeepMind’s publicly announced purposes for holding sensitive data on Royal Free’s patients, i.e. the management and direct care of AKI, were narrower than the purposes that contractually constrained its use of the data. These constraints were described in an eight page information sharing agreement (ISA) between Google UK Limited and Royal Free, signed on 29 September 2015 [4]. The Google-Royal Free ISA stated that, in addition to developing tools for ‘Patient Safety Alerts for AKI’ (presumably via the application now badged as Streams), Google, through DeepMind, could also build “real time clinical analytics, detection, diagnosis and decision support to support treatment and avert clinical deterioration across a range of diagnoses and organ systems” [10]. Further, it stated that the data provided by Royal Free was envisaged for use in the creation of a service termed ‘Patient Rescue’, “a proof of concept technology platform that enables analytics as a service for NHS Hospital Trusts”.

This was the entirety of the language in the ISA specifying the purposes for data sharing between Royal Free and Google over a two-year period ending 29 September 2017. (The ISA was superseded, prematurely, by a new set of agreements signed on 10 November 2016. Those agreements are beyond the scope of the present article and will be considered in future work.) At least contractually, the original ISA seemed to permit DeepMind to build systems to target *any* illness or part of the body. Further, the ISA contained no language constraining the use of artificial intelligence (AI) technologies on the data, meaning that DeepMind’s assurance that “for the moment there’s no AI or machine learning” was, and remains, rather less convincing than “but we don’t rule it out for the future” [9]. In mid-2016, the app’s online FAQs reiterated the same sentiment, adding that if artificial intelligence techniques are applied to the data in the future, this would be announced on the company’s website, and indicating that the company will seek regulatory approval under research authorization processes [11].

Another subject unaddressed in the ISA was the Google question: i.e. how data shared under the scheme would be cabined from other identifiable data stored by Google, given that Google was the signing party to the contract and that the company’s business model depends on monetizing personal data. DeepMind has made regular public assurances that Royal Free data “will never be linked or associated with Google accounts, products or services” [9, 12]. Problematically, these assurances appear to have been given little to no legal foundation in Google and DeepMind’s dealings with Royal Free,^3^ even if there is no reason to disbelieve the sincerity of their intent [13]. The reality is that the exact nature and extent of Google’s interests in NHS patient data remain ambiguous.

### Data, direct care and consent

It is important to note that, though the ISA provided Google with a broad set of purposes contractually, it did not displace various other legal, regulatory and ethical restrictions. A pertinent restriction is that medical information governance in the UK is geared around obtaining explicit consent from each patient whose identifiable data is passed to a third-party, when that third-party is not in a direct care relationship with the patient in question.^4^ Direct care is defined as “an activity concerned with the prevention, investigation and treatment of illness and the alleviation of suffering of an identified individual” [14].

The data that DeepMind processed under the Royal Free project was transferred to it without obtaining explicit consent from—or even giving any notice to—any of the patients in the dataset. For patients who had the necessary precursor renal blood test and were then progressed to being monitored by clinicians for AKI, the appropriate direct care relationship would exist to justify this data processing, through the vehicle of implied consent. However, the dataset transferred to DeepMind extended much more broadly than this. In fact, it included every patient admission, discharge and transfer within constituent hospitals of Royal Free over a more than five-year period (dating back to 2010). For all the people in the dataset who are never monitored for AKI, or who have visited the hospital in the past, ended their episode of care and not returned, consent (explicit or implied) and notice were lacking. This is an issue to which we will return, given the centrality of these requirements to patient privacy and meaningful agency.

On 29 April 2016, the extent of data held by DeepMind was revealed following a *New Scientist* investigation [15]. Google, which acted as the media filter for its subsidiary until at least October 2016, issued a swift public relations response. In all of its communications, Google insisted that it would not be using the full scope of the ISA it had signed [15], emphasizing that DeepMind was only developing an app for monitoring kidney disease [16]. This was despite the clear statements in the ISA quoted above, i.e. that information was also being shared for the development of real time analysis and alert systems, potentially as part of a broadly-defined ‘analytics as a service’ platform. On 4 May 2016, Royal Free issued a statement in line with Google’s position [17].

The data package described in the ISA and destined for DeepMind is patient identifiable, and includes the results of every blood test done at Royal Free in the five years prior to transfer [18]. It also includes demographic details and all electronic patient records of admissions and discharges from critical care and accident and emergency. It includes diagnoses for conditions and procedures that have a contributory significance to AKI, such as diabetes, kidney stones, appendectomies or renal transplants, but also those that do not, such as setting broken bones.

### A ‘national algorithm’ for AKI

Both DeepMind and Royal Free claim that Streams relies solely on a ‘national algorithm’ for AKI published by the NHS [19]; a process designed to assist in the rapid diagnosis of AKI from the starting point of a renal blood test for creatinine levels [20]. The implication is that all that Streams does is host this algorithm, and pump the Royal Free data (as stored, structured, formatted and delivered by DeepMind) through it to generate alerts [21, 11].^5^ These alerts are transmitted to a clinician’s mobile device, along with historical data on the patient in question to analyze trends (in seeming contradiction to the ISA, which stated that historical information was shared only “to aid service evaluation and audit on the AKI product”). Adding any new functions to the app, or fulfilling any of the broader contractual purposes described in the ISA, would comprise research. DeepMind did not have the requisite approvals for research from the Health Research Authority (HRA) and, in the case of identifiable data in particular, the Confidentiality Advisory Group (CAG) [22, 23]. Because DeepMind’s processes and servers—and those of the third-party datacenter holding the data—have not been independently scrutinized and explained, what the company has been, and is actually, doing with the data is not public.

The national AKI algorithm was launched in a patient safety alert put out by NHS England on 9 June 2014, recommending “the wide scale introduction and uptake of an automated computer software algorithm to detect AKI” [24]. The algorithm was standardized by a working group of nephrologists and biochemists, with inputs from providers of specialized laboratory software systems, and leads to results being generated in Royal Free’s laboratory information management system [25]. DeepMind’s asserted role has been to design a clinical app to get the alerts generated by this algorithm delivered to clinicians ‘on the fly’. The algorithm does not, however, extend to patients who have never been tested for serum creatinine, nor does it mention historical contextual data [26, 27]. It is only an assistant to good clinical care [28, 29], and its sensitivity and effectiveness remains a vibrant, contested field of research. As DeepMind has acknowledged, “the national algorithm can miss cases of AKI, can misclassify their severity, and can label some as having AKI when they don’t” [30]. The failure to explain and address these issues and, in particular, the disconnect between the Trust-wide dataset that has been transferred under the broad terms of the ISA and the narrower set of patients who will ever be monitored and treated for AKI, throws considerable doubt on the DeepMind-Royal Free position that all of the data being transferred is necessary and proportionate to the safe and effective care of *each* individual patient [31, 32].

## Grand designs and governance gaps

Between late April 2016, when the scale of the data transfer from Royal Free to DeepMind and the relative lack of constraints on its use became publicly known, and until at least October 2016, DeepMind and Royal Free maintained the narrative that the entire purpose of transferring millions of patient records was to assist with AKI diagnosis and alerts, under a relationship of direct patient care [33]. This position, however, fails to justify both the initial breadth of the data transfer and the continued data retention.

### Questioning direct care

Royal Free states that AKI affects “more than one in six in-patients” [17].^6^ If, as DeepMind claims, it only uses patient data in the service of monitoring and treating AKI, then it follows that as many as five sixths of patients (though this quantity is very unclear on the current state of the evidence) are not in a direct care relationship with the company. The distinction between being monitored or treated for AKI and not being monitored matters, because under British medical information governance guidelines [34], a direct care relationship between an identified patient and an identified clinical professional or member of a clinical care team obviates the need for explicit consent. Without such a direct care relationship, however, and without another basis such as consent, a formal research authorization from the HRA CAG, or otherwise satisfying necessity requirements and introducing appropriate safeguards [35], it is unlawful to continue to process patient data under the UK Data Protection Act 1998 (DPA).

As already noted, DeepMind has held data on millions of Royal Free patients and former patients since November 2015, with neither consent, nor research approval. The company, with the support of Royal Free, has elected to position itself as having a direct care relationship, by virtue of its AKI alert app, with each and every one of those patients. Drawing boundaries around the patients who are in a direct care relationship is not likely to be as clean as saying that it extends only to those who contract AKI, since the purpose of the app also includes monitoring. However, since the large, Trust-wide group whose data has been transferred includes individuals who have never had a blood test, never been tested or treated for kidney injury, or indeed patients who have since left the constituent hospitals or even passed away, the position that Royal Free and DeepMind assert—that the company is preventing, investigating or treating kidney disease in every patient—seems difficult to sustain on any reasonable interpretation of direct patient care.

### Grand ambitions and systemic change

Despite the public narrative’s exclusive focus on AKI, it is clear that DeepMind and Royal Free have always had designs on much grander targets. A memorandum of understanding (MOU) between DeepMind and Royal Free was signed on 28 January 2016, though was only discussed for the first time in June 2016, after being uncovered by a freedom of information request [36]. The document, which is not legally binding, talks about plans for DeepMind to develop new systems for Royal Free as part of a “broad ranging, mutually beneficial partnership… to work on genuinely innovative and transformational projects” [37]. Quarterly meetings are envisaged for the setting of priorities on product development, including real time prediction of risks of deterioration, death or readmission, bed, demand and task management, junior doctor deployment/private messaging, and the reading of cardiotocography traces in labor [38]. Although the MOU states that all such projects will be governed by their own individual agreements, the initial Royal Free ISA already covers DeepMind for the development of a wide range of medical tools.

These are vast ambitions, considerably out of step with DeepMind and Royal Free’s narrow public relations orientation towards their collaboration being entirely founded on direct care for AKI. The MOU also makes apparent the esteem in which DeepMind is held by its public service partners, indicating in the principles under which the parties intend to cooperate that one of the major reasons for Royal Free’s desired collaboration is “Reputational gain from a strategic alliance with an unrivalled partner of the highest profile and expertise, focused on a highly impactful mission”, plus a “place at the vanguard of developments in … one of the most promising technologies in healthcare”. DeepMind, by contrast, is in it for rather more sombre reasons: a clinical and operational test-bed, a strategic steer in product development and, most of all, for *data* for machine learning research.

Nascent indications of DeepMind’s plans for datasets that not only span a large healthcare trust such as Royal Free, but the entire NHS, have not yet received critical discussion [39], but can be seen in presentations given throughout 2016 by DeepMind cofounder and health lead, Mustafa Suleyman. These presentations have elaborated a vision for a “truly digital NHS”, comprising “massively improved patient care, actionable analytics, advanced research both at the hospital-wide level and the population-wide level, and an open innovation ecosystem” [40]. Suleyman characterizes this fourth element, underpinned technically by “digitizing most of the data that’s exchanged across the [NHS] system, open standards, and true interoperability”, as “the key pivotal thing” “that will enable us to bring a wide variety of providers into the system and for clinicians up and down the country to be able to commission much smaller, more nimble, startup-like organizations to provide some of the long tail of specialist and niche applications that nurses and doctors are asking for” [40]. At the core of Suleyman’s described vision is the “secure and controlled release of data” from what he terms “a single, back-end canonical record” that indexes, but also gives a degree of control to, all patients [41]—a telling sign of where a trust-wide dataset, retrofitted in a way that allows it to be leveraged by Google/DeepMind products and those of other technology companies, might ultimately be directed.

These statements are considerably broader than DeepMind and Royal Free’s public relations focus on the Streams AKI app, with very extensive implications deserving of full and rigorous consideration. As Suleyman describes it, the “very specific” targeting of AKI under Streams precedes a “real opportunity for us to go much much further and extend this to a broader patient-centric collaboration platform” [41]. Part of how this would be achieved technically, he indicated, was by making patient health data repurposable through an application programming interface termed FHIR (Fast Healthcare Interoperability Resources; pronounced ‘fire’); an open, extensible standard for exchanging electronic health records. The FHIR API, Suleyman indicated in July 2016, allows “aggregating the data in the back-end despite the fact that it is often spread across a hundred plus databases of different schemas and in different standards and in many hospitals”. He continued, “this is actually very tractable… it’s not a research problem, and we’ve actually had some success in starting to think about how we might do that, with the Royal Free” [41].

By September 2016, Suleyman was pitching DeepMind at the heart of a new vision for the NHS––and casting the Google-Royal Free collaboration in the terms that Google and DeepMind had vigorously denied and critics had feared (i.e. something much broader than an app for kidney injury, giving Google and DeepMind undue and anticompetitive leverage over the NHS [15]), highlighting sharply DeepMind’s unsatisfactory and quite possibly unlawful processing and repurposing of Trust-wide Royal Free data. Speaking at Nesta’s annual FutureFest, Suleyman stated: “Earlier this year, in February, we launched our first business that’s facing outwards, looking at how we can deploy our technologies to radically transform the NHS, digitize, and then help better organize and run the National Health Service [42].” DeepMind’s website pertaining to Streams was also updated, to state “We’re building an infrastructure that will help drive innovation across the NHS, ultimately allowing clinicians, patients and other developers to more easily create, integrate and use a broad range of new services” [43].

### Riding high above regulatory streets

When Royal Free transferred millions of patient records to DeepMind in November 2015, it was done without consulting relevant public bodies. The UK has an Information Commissioner’s Office (ICO), responsible for enforcing the Data Protection Act. The Health Research Authority (HRA) provides a governance framework for health research, and provides a path for the release of confidential health information in the absence of explicit consent, through the Confidentiality Advisory Group (CAG). The Medicines and Healthcare products Regulatory Agency (MHRA) regulates medical devices. None of these bodies were approached about the November 2015 data transfer [44]: not for informal advice from the ICO; not to go through an official and required device registration process with the MHRA before starting live tests of Streams at Royal Free in December 2015 [44]; and not to go through the HRA’s CAG, which could have been a vehicle for legitimizing many aspects of the project [45]. (DeepMind has subsequently been in discussion with all of these parties in reference to its Royal Free collaboration and, for several months from July 2016, stopped using Streams until the MHRA-required self-registration process was completed. [46] )

Instead, the parties went through just one third-party check before transferring the data: the ‘information governance toolkit’ [47], a self-assessment form required by NHS Digital (formerly HSCIC) [48], designed to validate the security of the technical infrastructure DeepMind would be using [49]. The same tool has been used for self-assessment by some 1500 external parties. The tool assists organizations to check that their computer systems are capable of handling NHS data, but it does not consider any of the properties of data transfers such as those discussed in this paper. NHS Digital conducted a routine desktop review of DeepMind’s toolkit submission in December 2015 (after data had been transferred) and approved that the third-party datacenter contracted by Google had adequate security [50]. Beyond this surface check, NHS Digital made no other enquiries. It subsequently confirmed the security of the external datacenter with an on-site check, but it was beyond the scope of NHS Digital’s role to assess the flow of data between Royal Free and Google or to examine any other parts of Google or any aspect of the data sharing agreements [50].

While the DeepMind-Royal Free project does have a self-assessed Privacy Impact Assessment (PIA) [51], as recommended by the ICO [52], the assessment commenced on 8 October 2015 [53], only *after* the ISA was signed, i.e. once the rules were already set. The PIA also failed to give any consideration to the historical data trove that was transferred under the ISA, as well as omitting to discuss privacy impacts on patients who never have the requisite blood test or otherwise proceed through the AKI algorithm that Streams uses, but whose data is in DeepMind’s servers, and which is formatted, structured, and prepared for repurposing anyway. That is to say, it neglected to deal with the primary privacy issues, as well as to justify the failure to address basic data processing principles such as data minimization. At the time of publication, the ICO was investigating the data transfer (primarily on whether data protection law requirements have been satisfied) [54], as was the National Data Guardian (primarily on the adequacy of the ‘direct care’ justification for processing) [55]. The only remaining health regulator in the picture is the Care Quality Commission (CQC), which gave a statement in October 2016 indicating the CQC would consider reported data breaches to the ICO as part of its own inspections, but otherwise declined to comment on the data transfer, indicating that it was broadly supportive of experimentation with big data-based care solutions “if they will lead to people getting higher quality care without undermining patient confidentiality” [56].

One year after data started to flow from Royal Free to DeepMind, the basic architecture of the deal had not visibly changed. On the other hand, subsequent deals between DeepMind and other London medical institutions, this time for research rather than direct patient care, were announced in a way that avoided many of the same questions. In these arrangements, data was anonymized before being transferred to DeepMind, and research approval (which raises separate issues, as discussed further below) was sought and gained before any research work commenced. Crucially, DeepMind and its partners were clear about the purposes and amount of data that would be transferred in those deals.

## Assessing the damage

The most striking feature of the DeepMind-Royal Free arrangement is the conviction with which the parties have pursued a narrative that it is not actually about artificial intelligence at all, and that it is all about direct care for kidney injury—but that they still need to process data on all the Trust’s patients over a multi-year period. This is hardly a recipe for great trust and confidence, particularly given that the arrangement involves largely unencumbered data flows, both with one company, DeepMind, whose *raison d’être* is artificial intelligence; and its parent, Google, the world’s largest advertising company, that has long coveted the health market [57]. Combined with the unavoidable fact that a sizeable number of patients never need care for kidney injury, the absence of any public consideration of patient privacy and agency, and the lack of safeguards to prioritize public goods and interests over private ones, there are reasons to see the deal as more damaging than beneficial.

Large digital technology companies certainly have the potential to improve our healthcare systems. However, given the sensitivity of building public trust in emerging technology domains, in order for innovation to deliver over the long-term, it must advance in a way that meets and exceeds existing regulatory frameworks and societal expectations of fair treatment and value. Not doing so will only hinder the adoption and growth of beneficial technology.

In this section, we identify a number of salutary lessons from the case study, assessing their implications for DeepMind, in particular, and for current and future directions in healthcare, more generally. These lessons draw on the themes of consent, transparency, privatization and power. Our ambition is to span from the details presented in the previous two sections towards the broader dynamics at play, both in the present deal and in the longer-term ambitions of AI-driven medical tools. The DeepMind-Royal Free deal is fast being converted from an optimistic mistake into a long-term partnership. What are the implications, both for this deal and for others that loom?

The significance of this case study is not only that there are retrospective and grave concerns about the justifiability of DeepMind’s continued holding of data on millions of citizens. The case study also offers a prism on the future. It offers one angle into how public institutions and the public at large are presently equipped to grapple with the promised rise of data-driven tools in domains such as medicine and public health. And it tests our assumptions and responses to Google/Alphabet and other speculative private prospectors of this algorithmic age—‘New Oil’, ‘New Rail’, ‘New Pharma’, we could say––as they transition from web-based markets, into land- and body-based markets.

### The conversation we need

It was only after an independent journalistic investigation revealed the necessary information—seven months after DeepMind and Royal Free first entered into a data sharing agreement, five months after the data had been transferred into DeepMind’s control and during which product development and testing had commenced, and two months after the project had been publicly announced––that *any* public conversation occurred about the nature, extent and limits of the DeepMind-Royal Free data transfer. Despite the shortcomings in the deal’s structure, if DeepMind and Royal Free had endeavored to inform past and present patients of plans for their data, initially and as they evolved, either through email or by letter, much of the subsequent fallout would have been mitigated. A clear lesson of this whole arrangement is that attempts to deliver public healthcare services should not be launched without disclosing the details, documentation, and approvals—the legal bedrock—of the partnerships that underlie them. This lesson applies no less to companies offering algorithmic tools on big datasets than it does to pharmaceutical and biotech companies.

The failure on both sides to engage in any conversation with patients and citizens is inexcusable, particularly in the British context, in the wake of the 2013 Caldicott review into information governance practices [34], the very public and profoundly damaging 2013–15 failure of the government’s care.data data sharing scheme [58], the 2014 recommendations of the National Data Guardian in the wake of the care.data debacle [59], and the 2015 Nuffield Council report on bioethics [60]. The clear take-away from these reports and recommendations––and indeed the entire regulatory apparatus around healthcare––is that patients should be able to understand when and why their health data is used, with realistic options for effective choice [61]. Patients should not be hearing about these things only when they become front-page scandals [62].

The DeepMind-Royal Free data deal may be just one transaction, but it holds many teachings. To sum up:

1. We do not know––and have no power to find out––what Google and DeepMind are really doing with NHS patient data, nor the extent of Royal Free’s meaningful control over what Google and DeepMind are doing;
2. Any assurances about use of the dataset come from public relations statements, rather than independent oversight or legally binding documents;
3. The amount of data transferred is far in excess of the requirements of those publicly stated needs, but not in excess of the information sharing agreement and broader memorandum of understanding governing the deal, both of which were kept private for many months;
4. The data transfer was done without consulting relevant regulatory bodies, with only one superficial assessment of server security, combined with a post-hoc and inadequate privacy impact assessment;
5. None of the millions of identified individuals in the dataset were either informed of the impending transfer to DeepMind, nor asked for their consent;
6. The transfer relies on an argument that DeepMind is in a “direct care” relationship with each patient that has been admitted to Royal Free constituent hospitals, even though DeepMind is developing an app that will only conceivably be used in the treatment of one sixth of those individuals; and
7. More than 12 months into the deal being made, no regulator had issued any comment or pushback.

If account is not taken of these lessons, it could result in harms beyond the breach of patients’ rights to confidentiality and privacy––though these elements in themselves should be enough to demand a regulatory response. Some of the potential risks posed by unregulated, black box algorithmic systems include misclassification, mistreatment, and the entrenchment and exacerbation of existing inequalities. It does not take an active imagination to foresee the damage that computational errors could wreak in software applied to healthcare systems. Clearly, the same skills and resources must be devoted to the examination and validation of data-driven tools as to their creation.

Without scrutiny (and perhaps even encouraged competition) Google and DeepMind could quickly obtain a monopolistic position over health analytics in the UK and internationally. Indeed, the companies are already in key positions in policy discussions on standards and digital reform. If a comprehensive, forward-thinking and creative regulatory response is not envisaged now, health services could find themselves washed onwards in a tide of efficiency and convenience, controlled more by Google than by publicly-mind health practitioners. Aggregating and centralizing control of health data and its analysis will generate levers that exist beyond democratic control, with no guarantees except for corporate branding and trust as to where they might end up.

It is important to reflect on these scenarios not as a prediction of what will come to pass, but as a vision of the potential danger if policymakers and regulators do not engage with digital entrants such as DeepMind and incumbents such as Google. There may be other, worse outcomes. To demand that innovation be done in a principled manner is not to stand in its way––it is to save it.

### Data protection

DeepMind and Royal Free have coalesced on the justification of direct care and implied consent to seek to justify the sharing of the Royal Free dataset [48, 17, 32]. Although this is the only available basis for them to justify the data transferred in November 2015, it sets a dangerous precedent for the future. To understand why, we need to step through the UK data protection and medical information governance frameworks.

Under the UK Data Protection Act, DeepMind needs to comply with a set of data protection principles, including having a legitimate basis at all times for processing information that can identify an individual [63]. Health information is classed as ‘sensitive personal data’ under this law [64], and is subject to additional safeguards [65]. For DeepMind, legitimate processing of health information comes down to one of two alternatives. Either it requires explicit consent from the individual concerned, which DeepMind does not have, or DeepMind must show that its processing is “necessary for medical purposes” (defined as “the purposes of preventative medicine, medical diagnosis, medical research, the provision of care and treatment and the management of healthcare services”) and “undertaken by (a) a medical professional; or (b) a person who in the circumstances owes a duty of confidentiality which is equivalent to that which would arise if that person were a health professional” [66].

Simply using health data for the purpose of speculative and abstract “medical purposes” does not satisfy data protection law. This is where the medical information governance architecture—the so-called Caldicott principles and guidelines [34]—come into play. Before turning to these rules, it is important to address an outstanding core issue in the data protection aspects of the Royal Free-DeepMind project.

Data protection law relies on a key distinction between ‘data controllers’ and ‘data processors’ [67]. A data controller is defined as “a person who (either alone or jointly or in common with other persons) determines the purposes for which and the manner in which any personal data are, or are to be, processed”, while a data processor is “any person (other than an employee of the data controller) who processes the data on behalf of the data controller” [68]. It is crucial to define controller and processor status in any information sharing arrangement because legal obligations and liabilities flow from it [69], with significant real-world consequences [70]. In essence, data controllers bear primary responsibility for complying with the principles of data protection, for accommodating data subject rights and other requirements, and are liable for damages in case of non-compliance.

The ISA between Royal Free and DeepMind states at a number of points that Royal Free is the data controller, while DeepMind is merely a data processor. While this is clearly the intention of the parties, the legal question is one of substance, not form. The substantial issue turns on applying the provisions of the DPA, particularly paragraphs 11–12 of Schedule 1, Part II. These provisions require, respectively, under paragraph 11 that a data processor provide sufficient guarantees and compliance in respect of the technical and organizational security measures governing the processing to be carried out and, under paragraph 12, that the processing be carried out under a contract, made or evidenced in writing, under which the processor “is to act only on instructions from the data controller”.

It seems clear that Royal Free have contracted with DeepMind to analyze complex data and come up with solutions by applying DeepMind’s own expertise in analysis to an extent that Royal Free cannot begin to do. Apart from the parties’ consensus on the overall purpose of processing––to assist in monitoring AKI using the nationally-mandated AKI algorithm––DeepMind seems to have considerable discretion, in addition to Royal Free, to determine the purposes and manner in which any personal data is processed. The company is storing, structuring and formatting the Trust-wide dataset, testing it, preparing to deliver data and visualizations to clinician’s devices and, most recently, discussing technical infrastructure that could enable it to be repurposed. These factors all point very strongly to DeepMind assuming the role of a joint data controller. Certainly, Royal Free, in its responses to investigations and freedom of information requests, has never provided any specific awareness or understanding of the means of DeepMind’s processing.

Further, even if DeepMind were to avoid the substantive factual conclusion that it is determining the purposes and manner of data processing, the document that is said to constrain DeepMind’s processing—the ISA—has a number of shortcomings that undermine its status as a ‘contract’ satisfying the mandatory requirements for data controller-processor relationships in Schedule 1, Part II, paragraph 12 of the DPA. The contract plausibly extends to a wide range of health tools for any health condition, without overriding controls from Royal Free. There is an absence of evidence in writing that DeepMind will act only on instructions from Royal Free, that data will not be linked with other datasets held by Google or DeepMind, or that the data will not be repurposed for other uses. It is irrelevant whether or not the parties would actually do any of these things. Assurances from the parties are not what matters here––what matters is what is stated in the document that purports to be the governing contract. Finally, the status of the document as a contract is diminished by its absence of any discussion of consideration passing between the entities.

DeepMind cannot be converted to being a pure data processor by having both parties sign an agreement declaring that this is its status, no matter how much the parties might wish it [71]. The situation is analogous to the example given by the ICO of a sharing agreement between a car rental service and a tracking company that helps ensure that cars are returned [70]. The agreement allows the tracking company to hold customer location data for a set period. However, the ICO states that because the tracking company applies its own secret knowledge in deciding the data to collect and how to analyze it, the fact that the rental company determines the overall purpose of tracking (i.e. car recovery) is not sufficient to make the tracking company a processor. Addressing and resolving the status of DeepMind is crucial, and is presumably a core dimension of the ICO’s ongoing investigations of the deal. In our assessment, it is clearly arguable that DeepMind is a joint data controller along with Royal Free. It is unfortunate that the ICO had not yet made a clear determination to resolve the question of legal status in over 12 months after the deal commenced, leaving individual rights and organizational responsibility hanging in the balance.

### Caldicott guidelines

The Caldicott rules help reduce the friction on data sharing of identifiable health information for direct patient care, while ensuring that other uses of such information—indirect care, such as research on identifiable individuals or risk prediction and stratification––are accorded sufficiently strong regard to legal obligations of privacy and confidentiality. In relation to Streams, the argument made by Google and Royal Free—and their only arguable basis for continuing to process the totality of data made available under the ISA—is that DeepMind is in a direct patient care relationship with all Royal Free patients. The assertion seems to be that, since any Royal Free patient may deteriorate with AKI in the future, the hospitals are justified in sharing the superset of everyone’s medical information with Google now, just in case a patient needs DeepMind’s services in the future. To this the odd claim is added that “with any clinical data processing platform it is quite normal to have data lying in storage” [72], without acknowledging necessary legal and ethical limits to such a claim. This line of reasoning is unsustainable if ‘direct care’ is to have any useful differentiating meaning from ‘indirect care’. By the same argument, DeepMind would be justified in holding all the data on every patient in the NHS, on the basis that one of those patients, one day, might require direct clinical treatment through Streams [73].

DeepMind’s situation has no clear direct analogy in the Caldicott guidelines. Usually when speaking of the implied consent inherent in direct care relationships, the guidelines describe scenarios where registered clinical professionals acting as part of a clinical team, all with a legitimate relationship with the patient, pass relevant patient data between themselves, e.g., a surgeon passing a patient to a post-operation nurse [34, 74]. Implied consent in these scenarios is easily justified. It builds on the core relationship between a patient and a clinical professional, within which tools—including software tools, record management systems, alert and analytics systems, etc.—can be introduced in the service of patient care. There are also safeguards, such as that complaints can be made to the General Medical Council.

For individuals who are escalated to clinical intervention based on the results of applying the AKI algorithm after a preliminary blood test, clearly this direct care scenario applies. However, for the remainder of patients whose data has been transferred to DeepMind, no plausible necessity for DeepMind’s processing of their data arises. It is, instead, a classic situation of health services management, preventative medicine, or medical research that applies to the overall provision of services to a population as a whole, or a group of patients with a particular condition. This is the very definition of indirect care [34]. Lawful processing of identifiable data for indirect care, if there is no consent, can only proceed under what is termed the ‘statutory gateway’––i.e. under section 251 of the NHS Act 2006 (UK) and The Health Service (Control of Patient Information) Regulations 2002. In effect, s.251 allows third-parties to bypass the impracticality of gaining consent from large numbers of patients to process their data, by asking the Secretary of State for Health on the patients’ behalf through the HRA CAG approval process. It is notable that the process that Royal Free and DeepMind assert is necessary here—of storing, structuring and formatting trust- or hospital-wide datasets in order to then effectively deliver clinical care to a subset of patients—does not naturally fall into any of the envisaged s.251 circumstances in which confidential patient information may be processed [75].

A final element in addition to the hard legal arguments about consent and the ICO, and direct care and the Data Guardian, is notice. Notice is not a mandatory requirement under the DPA if data is lawfully repurposed, but it is necessary if data is being processed for a new purpose [76]. This would be the case, at the very least, for patients who are in the transferred dataset, but who are never tested and treated for kidney injury. Though at the broadest level Royal Free is engaged in repurposing data acquired for medical purposes, in this case, to argue that this data is legitimately being repurposed *en masse* in the DeepMind deal undermines wholly the protections afforded to such sensitive data in both the DPA and the Caldicott rules.

As a partial acknowledgment of this, in May 2016 Royal Free highlighted that an opt-out exists to data sharing with Google/DeepMind [17]. However, the opt-out was only made clear after public attention had been called to the deal. Such an after-the-fact concession strikes as poor compensation, and is inconsistent with the practice of other hospitals in endeavors of similar reach. Take for example the 2015 Connecting Care project, comprising Bristol, North Somerset and South Gloucestershire hospitals [77]. This project involved a more sound basis for population-wide data sharing based on implied consent, because it concerned various third-party providers being linked to provide an electronic patient record system. A mass mailing of information on the parties involved, and reasons for data processing, to all individuals in the community was undertaken as a key exercise to inform and allow individuals to opt out, and was followed up with ongoing efforts to inform patients. Though this project was more involved than the Royal Free-DeepMind deal, it also had a more legitimate reason for extending across the entire population of constituent hospitals. Royal Free has not justified why a similar process did not take place with its arrangements with Google.

Given Streams is characterized as a clinical app, there are more elegant––and less legally and ethically dubious––solutions available than simply running a mirror copy of the Royal Free’s repository of patient data on third-party servers controlled by DeepMind, for every single hospital patient, entirely independently of AKI susceptibility and diagnosis. One solution is for DeepMind to pull in historical data only on patients who have had the gateway blood test that is prerequisite for AKI diagnosis. If Royal Free’s systems cannot currently handle real time data requests in this manner, they ought to. It seems in the essence of an ethical and legal streaming service that just as a patient’s relevant blood tests from Royal Free ‘stream’ to DeepMind’s servers, so should historical data on the identified at-risk patients.

Below, we unpack the implications of these points with a focus on transparency, data value, and market power. There has been an inexcusable institutional delay in the NHS, ICO and Data Guardian’s response to the issues discussed so far. The remainder of this section exposes how ill-equipped our institutions are to deal with the challenges ahead.

### Transparency and the one-way mirror

At the heart of this deal is a core transparency paradox. Google knows a lot about all of us. For millions of patients in the Royal Free’s North London catchment, it now has the potential to know even more. Yet, when the tables are turned, we know very little about Google. Once our data makes its way onto Google-controlled servers, our ability to track that data––to understand how and why decisions are made about us––is at an end. Committed investigative reporting has led to documentation describing the DeepMind-Royal Free data transfer being made public, but we still have no real knowledge of what happens once the data reaches DeepMind, nor many tools to find out.

The public’s situation is analogous to being interrogated through a one-way mirror: Google can see us, but we cannot see it [78, 79]. The company benefits from relying on commercial secrets and the absence of public law obligations and remedies against it. This leaves it with few incentives for accountability. Only when it collides with institutions that have obligations to account—i.e. when it makes data sharing arrangements with Royal Free, or it applies for approval to NHS Digital––do rules such as the UK Freedom of Information Act 2000 permit some cracks in the glass.

This particular case study, and the way that it has unfolded, demonstrates the clear absence of strong tools to require companies to account in the same way as public institutions—even if they aspire to deliver, and in some cases even overtake, public services. There are many parallels to another contemporary policy issue involving Google: its application of a 2014 European court ruling requiring the company to delist information that is retrieved on name searches from its search engine when that information is not of public interest and is shown to have lost relevance, accuracy or timeliness [80]. In that case too, the one-way mirror has conceded only cracks of knowledge. The tools of discovery, to inform the public about privately-run services with deep impacts on their lives, are vastly unequal to the power that Google wields.

### Corporate responsibility

Even without portholes through which to examine the operations of powerful technology companies in detail, there is still a lot more that can be done, both from corporations themselves, and from the institutions that are mandated to oversee them. The deal-making between DeepMind and public institutions continues to be secretive. This is inappropriate for a system that typically requires open tender and disclosure. The purpose and terms of these deals should be made transparent, before committing populations of millions to them. They should clearly lay out the public benefit of their works, as well as the private benefits—what is in it for Google, for DeepMind? What initiatives have been made towards ensuring ongoing and equitable benefit-sharing? How are procurement rules and restrictions satisfied? While total transparency of processes is not possible, transparency of purpose and means must be—legitimizing, in detail, the company’s reasons and limits in holding sensitive data. To its credit, DeepMind’s announcement of deals subsequent to Royal Free have moved in this direction; although peer reviewers still question issues of consent [81], and the lack of details around the algorithmic processes to be applied [82].

DeepMind has taken steps towards self-regulation. When DeepMind announced Streams in February 2016, it also announced the creation of a board of what it termed ‘independent reviewers’––nine prominent public figures in the fields of technology and medicine in the UK—to scrutinize the company’s work with the NHS [83, 84]. The board met for the first time in June 2016. The board is ostensibly reviewing DeepMind’s activities in the public interest, but as at the end of January 2017, it had not made any minutes or account of its discussions public, nor had any reviewers expressed any concerns about DeepMind’s arrangements publicly. Annual statements are envisaged. Oversight of artificial intelligence as it is applied to healthcare is obviously desirable. But a self-appointed oversight board, arguably paid in the currency of reputational gain by association with successful technology companies, is far from adequate in itself. Being hand-chosen by DeepMind, the members of the board are unlikely to have positions fundamentally at odds with the company. It would also be a considerable about-face to denounce the whole arrangement with a partner such as Royal Free. At best, the board will supplement institutional external oversight mechanisms and provide insights not readily gained by outsiders: for example, access to internal data; independent assessments of internal arrangements for data handling, privacy and security; empirical insights into the attitudes of employees and the protection of the public interest. At worst, however, such a board risks creating a vacuum around truly independent and rightly skeptical critique and understanding.

The question of how to make technology giants such as Google more publicly accountable is one of the most pressing political challenges we face today. The rapid diversification of these businesses from web-based services into all sorts of aspects of everyday life—energy, transport, healthcare—has found us unprepared. But it only emphasizes the need to act decisively.

Machine learning tools offer great promise in helping to navigate complex information spaces, environments and work flows that are beyond the reach of any one clinician or team. However, it is essential that the supply chain of data and humans leading to any machine learning tools are comprehensible and queryable. This is a check on the impulse of technology startups that want to ‘move fast and break things’. While there is little doubt that individuals at DeepMind do care about improving the situation at Royal Free and across the NHS generally, the young company is clearly interested in moving fast—as are Royal Free’s clinicians. ‘The faster we move, the more lives we can save’, goes the logic. This may be true, but it injects several elements of dangerous risk, and potentially hazardous breakages, in developing these new tools: first, that the tools will provide misleading and harmful advice in edge cases; and second, that public trust and confidence in artificial intelligence erodes, making it harder to carry out projects in the future in sensitive areas, despite their promised benefits. Aligning the development and operation of artificial intelligence products with human-scale accountability and explanation will be a challenge. But the alternative is to abdicate ourselves to systems that, when they break, will not explain themselves to us.

It is worth noting that in digesting our medical records and histories, machine learning systems have the potential to uncover new hypotheses and trends about us, as a population, that are difficult to adapt to and deal with. It may turn out, for instance, that certain kinds of people are particularly susceptible to requiring an expensive medical intervention over the course of their lives. Regulations should require that the burdens of new discoveries not fall solely on the shoulders of those individuals who happen to need the intervention. There is a risk that, if we do not understand how companies like DeepMind draw knowledge from our data, we will not be prepared for the implications of the knowledge when it arrives.

It is essential that society is prepared for these newfound patterns, and able to protect those people who find themselves newly categorized and potentially disadvantaged. This newfound understanding of our condition will leave us all better off, but only if we share the burdens that the discoveries will place on individuals.

### Privatization and value for data

Even if DeepMind had been more open about its Royal Free data deal, as it was in subsequent research deals, questions still remain about the value that flows to the British public from these deals. DeepMind has made public two other partnerships with the NHS, both—unlike with Royal Free—for research rather than patient care, with actual involvement of AI, and with appropriate research approvals. One, with Moorfields Eye Hospital in London [85], involves the AI company receiving one million anonymized eye scans which it will run through its machine learning algorithms in search of new patterns of degeneration that might allow disease to be caught earlier [86]. Like the Royal Free collaboration, it commenced in July 2015 [87], when a Moorfields ophthalmologist approached DeepMind with a research question: can deep learning be used to diagnose age related macular degeneration or diabetic retinopathy? Approval to work on anonymized data was granted by Moorfields in October 2015 and the first part of an approval to work on pseudonymized data came in June 2016, at the same time as a research protocol was also published in an open access journal [88]. Ethical approval was granted, but it is worth noting that it was confined to looking at the risk of adverse patient events, not at broader questions such as the future for jobs, for competition, human deskilling, etc [82].^7^ The Moorfields project was announced publicly in July 2016. While other hospitals and startups can pursue similar projects, Moorfields sees more patients a year than any other eye hospital in the US or Europe.

The second partnership, with UCL Hospitals NHS Foundation Trust, sees DeepMind receiving 700 anonymized radiography scans [89]. The AI company is attempting to improve how treatment is planned for head and neck cancer, by speeding up scan segmentation––the process of deciding where and how to direct radiation in order to maximize impact to cancerous cells and minimize harm to healthy tissue. At the moment an expert radiologist needs to label images pixel-by-pixel, with a 28 day wait-time, for a four hour process [40]. DeepMind received approval to work on anonymized data in April 2016, with its research protocol published August 2016 [90].

The assumption is that DeepMind’s technical capability will let it discover new things about analyzing medical imagery, and that those new modes of analysis will be shared back to the community. However, documents binding DeepMind’s agreement with Moorfields and UCL, and the terms of data sharing, were not public as at October 2016. We do know that DeepMind will keep all algorithms that are developed during the studies. In other words, the knowledge DeepMind extracts from these public resources will belong exclusively to DeepMind. Even if it publishes the scientific results of its studies, it is unlikely it will freely publish the algorithms it has trained. In effect, the chance to train and exploit algorithms on real-world health data is DeepMind’s consideration for these deals. The consideration for its partners is that those algorithms––and the promise that they advance the field of diagnostics––exist in the world. Given this, the opacity of consideration passing between the parties in this, as with the contract with Royal Free, is problematic. There are no details on the clinical service and cost of any service to be provided by DeepMind in exchange for the data access, only vague statements that have been made in public fora about the possibility of a future levy being imposed, in alignment with improvements in clinical outcomes.

### Open competition and public interest

Offering DeepMind a lead advantage in developing new algorithmic tools on otherwise privately-held, but publicly-generated datasets limits the adoption of any scientific advances the company may make to two channels: via DeepMind on DeepMind’s terms; or to recreating, at expense and with unclear routes to access, DeepMind’s training on the same datasets.

Concepts of the value of data have not yet permeated popular culture. Google and other technology companies know very well what value they can unlock from a particular dataset and from access to millions or billions of computers that stream data on how their human owners walk, talk, travel and think.

But the public, and by extension the public sector, do not yet contemplate the value of this commodity that only they are capable of creating. Without people, there is no data. Without data, there is no artificial intelligence. It is a great stroke of luck that business has found a way to monetize a commodity that we all produce just by living our lives. Ensuring we get value from the commodity is not a case of throwing barriers in front of all manner of data processing. Instead, it should focus on aligning public and private interests around the public’s data, ensuring that both sides benefit from any deal [91].

The value embodied in these NHS datasets does not belong exclusively to the clinicians and specialists who have made deals with DeepMind. It also belongs to the public who generated it in the course of treatment. There is a pressing need for the NHS to consult broadly on the value-for-data aspects of these transfers, to ensure that the British public gets the best possible value out of any future deal. This value might take the form of an NHS stake in any products that DeepMind, a for-profit company, develop and sell using NHS data. It could be as simple as a binding agreement to share any future products with the entire NHS at a discount, or for free. It is inappropriate to leave these matters for future discussion, risking lock-in. There may even be scenarios where third-party processors can use NHS data to build products that are not related to health, but are useful in other markets. The public has a revulsion against ‘selling’ NHS data, but this impulse sells the public short on its own assets. The Royal Free-Google deal suggests that data will flow in any event, under the banner of innovation, without any value-for-money discussions. We recommend that, in addition to formalizing inputs on these aspects of value, the NHS might also consider the intrinsic impacts of automation [92]—how will clinicians interface with these new tools? How will the NHS deal with inevitable deskilling and shifts in the workforce, in response to automation? How will they ensure that the daily art of medicine is as protected and valued as the science?

A properly resourced and truly independent entity or entities should be tackling these challenges. Perhaps the Council of Data Science Ethics and standing Commission on Artificial Intelligence, recommended—and, in the first case, accepted by the government [93]—under two reports of the UK House of Commons Science and Technology Committee [94, 95], will be able to undertake this task, but their independence and rigor must be proven. They must also take into account the fact that DeepMind continues to rapidly expand its staff, including with senior appointments from the ranks of government and the NHS itself [96, 97].

### Market power

The new phenomenon of using machine learning to extract value from health data is likely the precursor of a general movement to monetize public datasets. Centralized government services are obvious targets for machine learning. They are directed towards fundamental human needs of care, housing, education and health, and often hold long baseline datasets about human behavior in areas where services could be improved. The complexity and scale of this information is what has led to the suggestion that these are areas where the sheer force of computation and algorithmic learning on large volumes of data offers great utility and promise.

When private companies access these resources with the intention of building on top of them, first-mover advantage exists as it does whenever private companies exploit public resources—land, fossil fuel stores, connection points to people’s homes. In the new realm of machine learning, it is important to ensure that DeepMind’s algorithms do not put it in an entrenched market position.

Of course, DeepMind is not the only innovator making overtures to the NHS, and machine learning is not the only innovation. In the case of kidney injury, outcomes would be as well influenced by employing more nurses to ensure that patients are hydrated, as deploying new algorithms. Some healthy caution about the first-mover is advised. If our public services have not laid the groundwork for an open, flourishing future innovation ecosystem, then the temptation for players like DeepMind to sit on their entrenched networks will be too strong.

It is important to note that, while giving DeepMind access to NHS data does not in principle preclude the same access being given to other companies in future, the willingness to recreate work, and ability to catch up, will diminish over time. Already, anecdotally, startups are reluctant to move in places where DeepMind has started deploying its immense resources. The danger of unconstrained, unreflective allocation of datasets to powerful parties is that the incentives for competition will distort. Like physical networks of electricity cables or gas pipes, it is perfectly possible for another company to redo what has been done by another. However, there are powerful inefficiencies and network effects that count against such possibilities. If we are to see the true promise of artificial intelligence, a much more positive solution would be to heavily constrain the dataset and to introduce a competitive, open process for simultaneous technology development by a range of private, public, and private-public providers.

A way of conceptualizing our way out of a single provider solution by a powerful first-mover is to think about datasets as public resources, with attendant public ownership interests. Ownership in this context is often a loaded notion, but it does not need to reduce to something that is atomized and commoditized for control at the individual level. Learning from commons movements [98], trusted institutions and communities appear to be the best vehicles to advocate for individual rights, rather than placing the burden of ownership on individuals. The key then is to return value at the communal level [99]. Indeed, data held by NHS trusts ought to be perfectly positioned for this treatment.

Hospitals are a community dedicated to the care of their patients. The first step for DeepMind and Royal Free should have been to engage the community in explaining the solutions they will pursue, and achieving buy-in with communal control and reward. The second step would have been to expand this with other alternatives in a flourishing innovative ecosystem. This did not happen, and it does not look like it will happen. In this regard, it is important to note that offering functionality for patients to see and audit their own data as it moves through systems [100, 101], as DeepMind has intimated that it will do in the future, is a positive development, but it is also one that resigns itself to perpetuating ultimate control, and a power asymmetry, in the hands of those who control the system—in this case, DeepMind. None of the approaches of DeepMind, of Google, or of the industry-supported Partnership on Artificial Intelligence that they announced in 2016, do anything to mitigate this control. They trumpet their own good intents, in benefiting the many in open, responsible, socially engaged ways that avoid undesirable outcomes [102]. But ultimately, these are tweaks within the frame of a certain deterministic approach to technology. They look for corporate initiative, not for robust solutions that stand outside our present paradigm and ask how best we can truly assure that we advance technologically, and that we do so in a way that ensures deep and broad public interests are met, not just superficially immediate, efficient, commercial solutions.

## Conclusion

The 2015–16 deal between a subsidiary of the world’s largest advertising company and a major hospital trust in Britain’s centralized public health service should serve as a cautionary tale and a call to attention. Through the vehicle of a promise both grand and diffuse––of a streaming app that will deliver critical alerts and actionable analytics on kidney disease now, and the health of all citizens in the future––Google DeepMind has entered the healthcare market. It has done so without any health-specific domain expertise, but with a potent combination of prestige, patronage and the promise of progress.

Networks of information now rule our professional and personal lives. These are principally owned and controlled by a handful of US companies: Google, Facebook, Microsoft, Amazon, Apple, IBM. New players cannot compete with these successful networks, whose influence deepens and becomes more entrenched as they ingest more data, more resources. If these born-digital companies are afforded the opportunity to extend these networks into other domains of life, they will limit competition there too. This is what is at stake with Google DeepMind being given unfettered, unexamined access to population-wide health datasets. It will build, own and control networks of knowledge about disease.

Fortunately, health data comes with very strong protections that are designed to protect individuals and the public interest. These protections must be respected before acceding to any promises of innovation and efficiency emanating from data processing companies. Public health services such as the British NHS are deeply complex systems. It is imperative for such institutions to constantly explore ways to advance technologically in their public health mission. Artificial intelligence and machine learning may well offer great promise. But the special relationship that has surged ahead between Royal Free and Google DeepMind does not carry a positive message. Digital pioneers who claim to be committed to the public interest must do better than to pursue secretive deals and specious claims in something as important as the health of populations. For public institutions and oversight mechanisms to fail in their wake would be an irrevocable mistake.

## Footnotes

1 Since Oct 2015, DeepMind has been owned by Google’s parent company, Alphabet Inc.

2 Given the time period considered, the EU’s General Data Protection Regulation 2016/679, which enters into force in May 2018, is beyond the scope of this article.

3 There is no prohibition on linkage in the ISA. DeepMind’s own internal privacy impact assessment (see section 3.3 below) states that no new linkages of data will be made, but this document has no legal force, and given its other shortcomings––i.e. that it does not deal with the bulk of the data transfer, nor the bulk of the individuals affected––we do not consider it adequate.

4 See discussion of consent in section 4.2-4.3 below.

5 The Streams FAQ states that “all development is done using synthetic (mock) data. Clinical data is used for testing purposes”.

6 This seems an upper estimate on the clinical reality: see [6].

7 As one reviewer remarked: “Overall a novel concept and worth exploring as it will be able to replace human workforce if successful”.
